# Supplementary material for: Chromatin swelling drives neutrophil extracellular trap release
Source: Nat Commun. 2018 Sep 14;9:3767. doi: 10.1038/s41467-018-06263-5 (PMC6138659; doi:10.1038/s41467-018-06263-5)
Supplement: Supplementary file 1 — Supplementary Information [file 41467_2018_6263_MOESM1_ESM.pdf]

Supplementary information

Chromatin swelling drives neutrophil extracellular trap release

Elsa Neubert[1,2*], Daniel Meyer[2,3*], Francesco Rocca[4], Gökhan Günay[1,2], Anja Kwaczala-Tessmann[1], Julia Grandke[1], Susanne Senger Sander[1], Claudia Geisler[3,4], Alexander Egner[3,4], Michael P. Schön[1,5], Luise Erpenbeck[1*+], Sebastian Kruss[2,3*+]

[1]Department of Dermatology, Venereology and Allergology, University Medical Center, Goettingen University, Germany

[2]Institute of Physical Chemistry, Goettingen University, Germany

[3]Center for Nanoscale Microscopy and Molecular Physiology of the Brain (CNMPB), Goettingen, Germany

[4]Optical Nanoscopy, Laser-LaboratoriumGoettingene.V., Germany

[5]Lower Saxony Institute of Occupational Dermatology, University Medical Center Goettingen and University of Osnabrück, Germany

*These authors contributed equally

[+]Correspondence should be addressed to L.E. or S.K. (email: luise.erpenbeck@med.uni-goettingen or skruss@uni-goettingen.de)

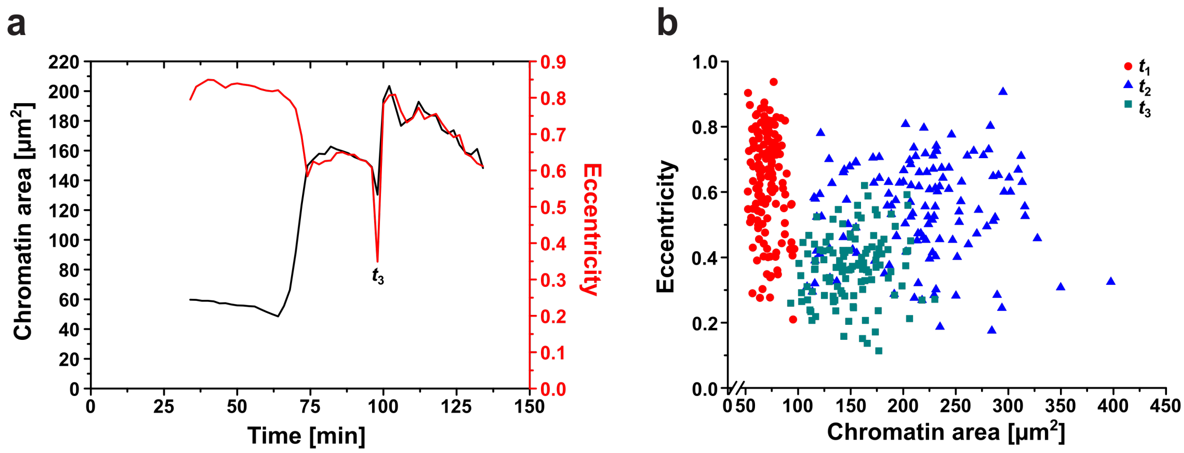

Supplementary Fig. 1: ***Eccentricity of chromatin during NETosis.*** *Chromatin rounds up during Phase 1 and 2 and reaches maximal circularity (minimal eccentricity) exactly before NET release (t_3).* ***a,*** *Eccentricity over time displayed for the example cell of* ***Fig. 1b*** *(**Supplementary Movie 2**).* ***b,*** *Comparison of the eccentricity at different time points (t_1, t_2, t_3) for in total 139 cells of five donors.*

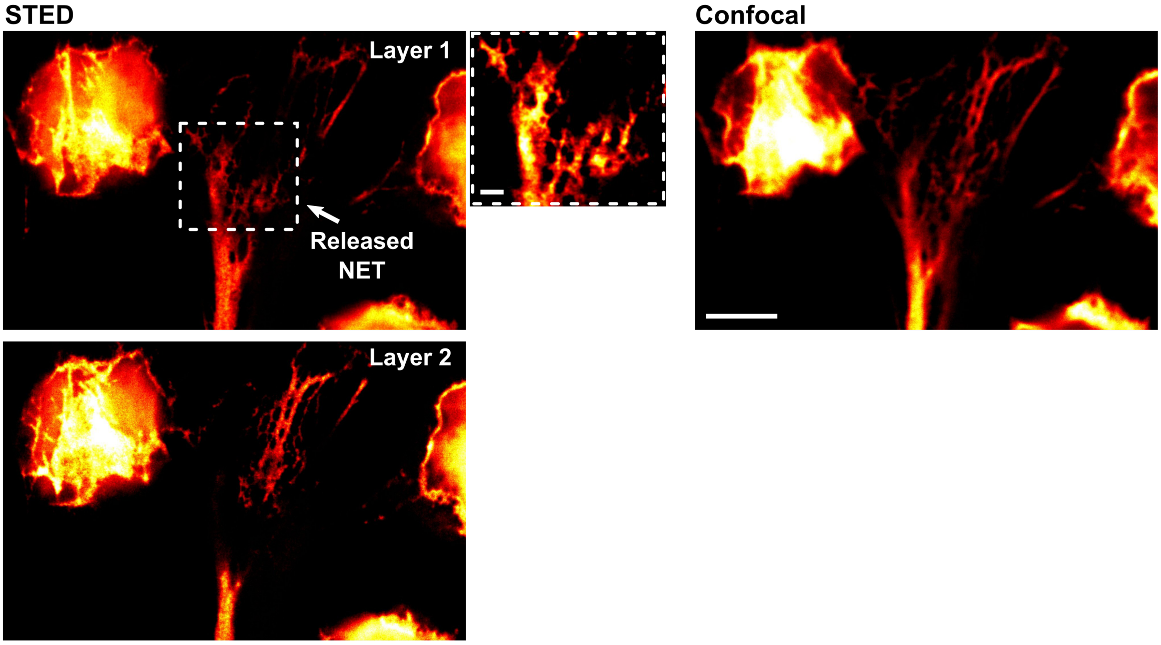

Supplementary Fig. 2: ***STED nanoscopy of NET fine structure.*** *Images of PMA-activated neutrophils (arrow = released NET) in comparison to confocal microscopy. Staining: SiR-DNA on fixed samples. Scale = 5 µm and 1 µm (insert).*

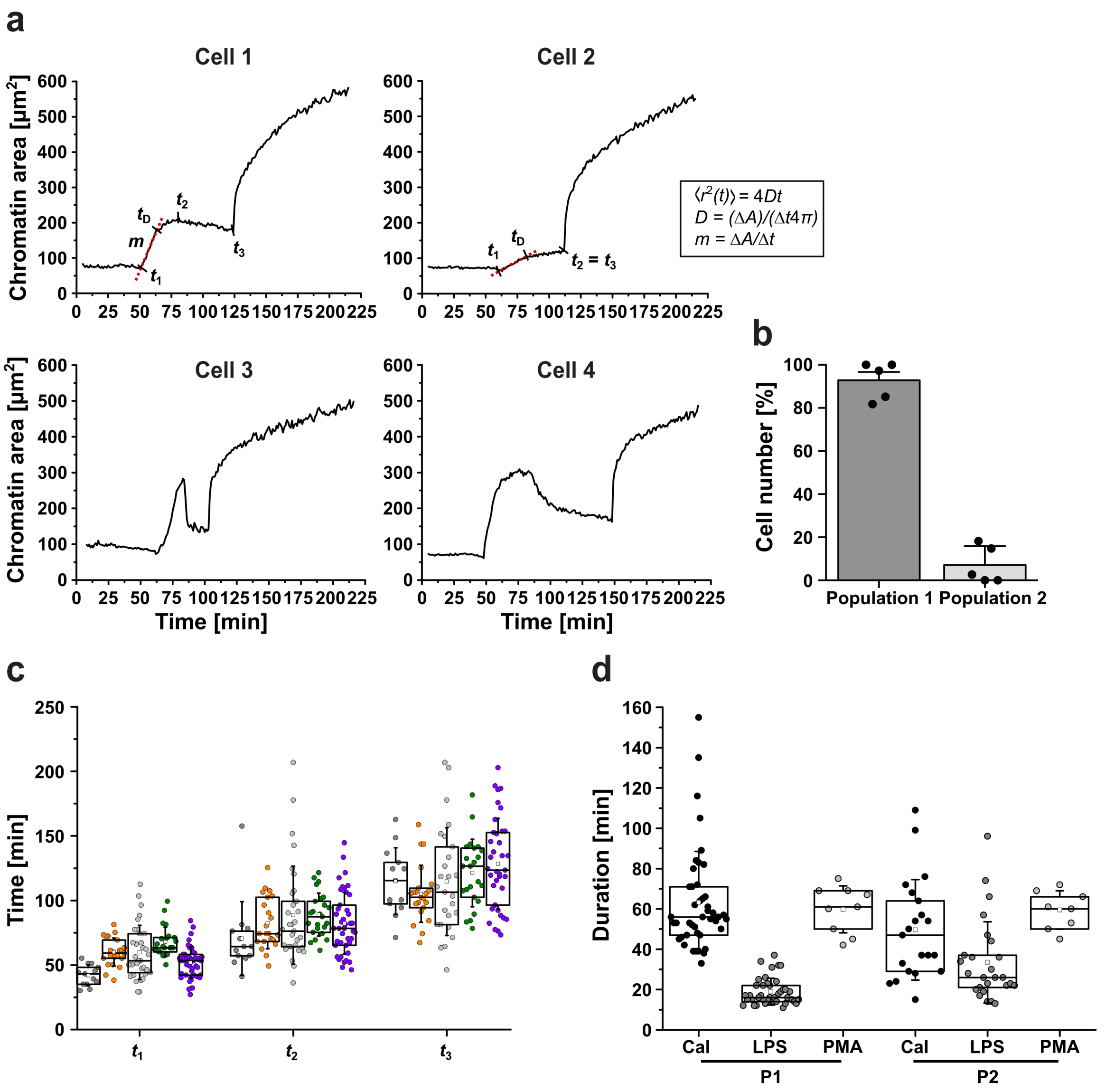

Supplementary Fig. 3: ***Characterization of phases of NETosis. a, b*** *Time course of changes in chromatin area during PMA-induced NETosis displayed for different cells imaged with wide field fluorescence microscopy. Cells can be divided in two different populations based on their chromatin time course. Cells of population 1 show a maximum of chromatin expansion (t_2) in P2. Chromatin of cells of population 2 expands until cell rupture at t_3. t_2 cannot be defined for this population (cell 2). Cell 1, 3 and 4 represent cells of population 1 and cell 2 of population 2. Therefore, only population 1 (> 90 % of cells) has been used to analyze the correlation between cell area at rupture/pressure P at t_2 and the time between t_2 and t_3 (rupture delay time) (**Fig. 4a, d**). The diffusion coefficient shown in **Supplementary Fig. S8a** is calculated based on the slope of the linear fit from t_1 to t_D. Mean ± SEM. **c,** Distribution of the time points t_1, t_2 and t_3 of all cells of five individual experiments (summary shown in **Fig. 1c**). Time points were calculated based on time-lapse movies recorded with conventional fluorescence microscopy. N = 5. **d,** Kinetics of NET formation after stimulation with three different stimuli (PMA = 100 nM, LPS = 25 µg ml^{-1}, CaI = 4 µM). Duration of P1 is significantly shortened after stimulation with LPS compared to PMA and CaI, whereas P2 displays only small stimulus dependence. N = 1. Boxplots (**c, d**) display the 25th and 75th percentile with the horizontal line at the median, squares represent the mean and whiskers the SD.*

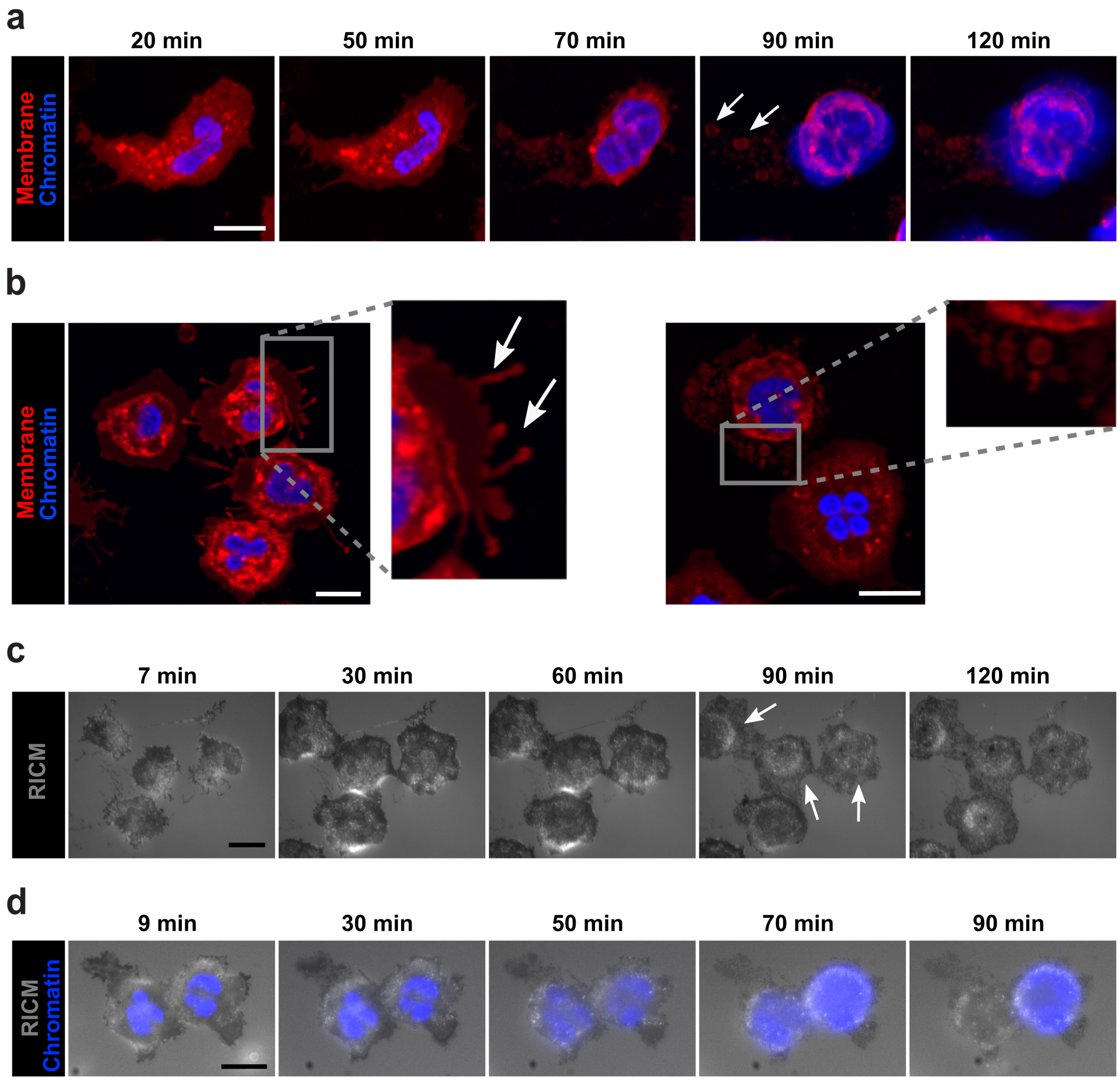

Supplementary Fig. 4: ***Membrane rearrangement during NETosis. a,*** *CLSM images of a human neutrophil undergoing NETosis (blue = Hoechst 33342/DNA, red = PKH26/membrane) observed by 3D-CLSM (see also* ***Supplementary Movie 8****). The cell spreads initially but retracts its cell body leaving membrane behind. Vesicles are formed closely bound to the substrate, probably originating from excess membrane (arrows, t = 90 min). Scale = 10 µm.* ***b,*** *Characteristic behavior of the cell membrane during NET formation observed by time-lapse CLSM (blue = DNA, red = membrane). Cells form membrane extensions (arrows) in late P1 (left) and membrane vesicles at the surface during the cell rounding process in P2 (right). Scale = 10 µm.* ***c,*** *Representative images of human neutrophils undergoing PMA-induced NET formation recorded with real-time reflection interference contrast microscopy (RICM) on glass. Images allow the label-free analysis of the cell/surface contact area (black = cell closer to the surface, white = further away from the surface). During NETosis, the cells round up, leave membrane closely bound to the substrate behind (arrows, t = 90 min) and expel the NET (see also* ***Supplementary Movie 9****). Scale = 10 µm.* ***d,*** *Overlay of real-time RICM with immunofluorescence (blue = chromatin) to verify NET release (t = 70, 90 min) during time-lapse RICM imaging. Scale = 10 µm.*

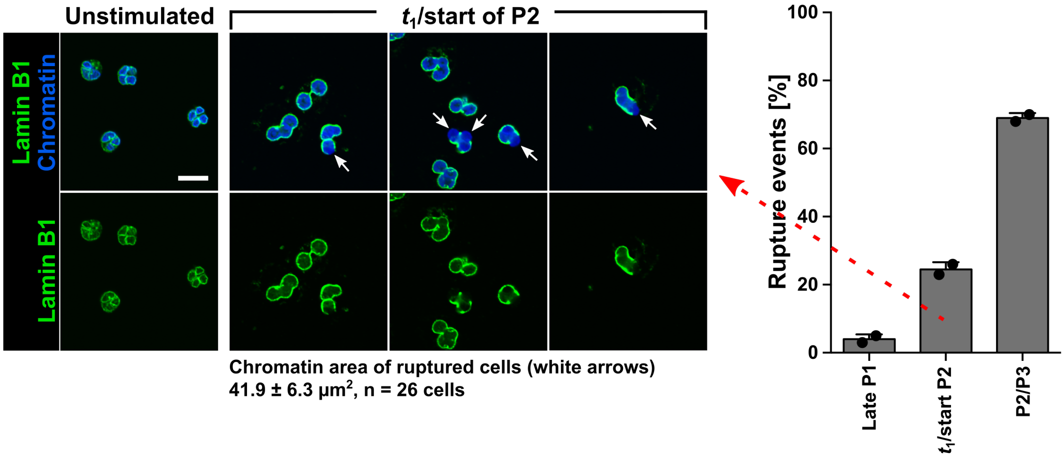

Supplementary Fig. 5: ***Rupture of the nuclear envelope at beginning of P2.*** *Overlay of chromatin (blue) and lamin B1 (green) of unstimulated and NETotic human neutrophil (CLSM, fixed samples). Average chromatin area at rupture point of the surrounding lamin B1 (t_1) is 41.9 ± 6.3 µm^2 (n = 26 cells from two donors) and in good agreement with the chromatin area at t_1 determined during live cell imaging (**Fig. 1a, b**). At this time point, the nuclear envelope rupture events increase (t_1/start P2) until a high rate of rupture events (small whole up to full loss of the Lamin B1 surrounding in P2 and P3). Calculation based on CLSM images of fixed samples. N = 2 donors (100 cells per condition and N). Mean ± SD. Scale = 10 µm.*

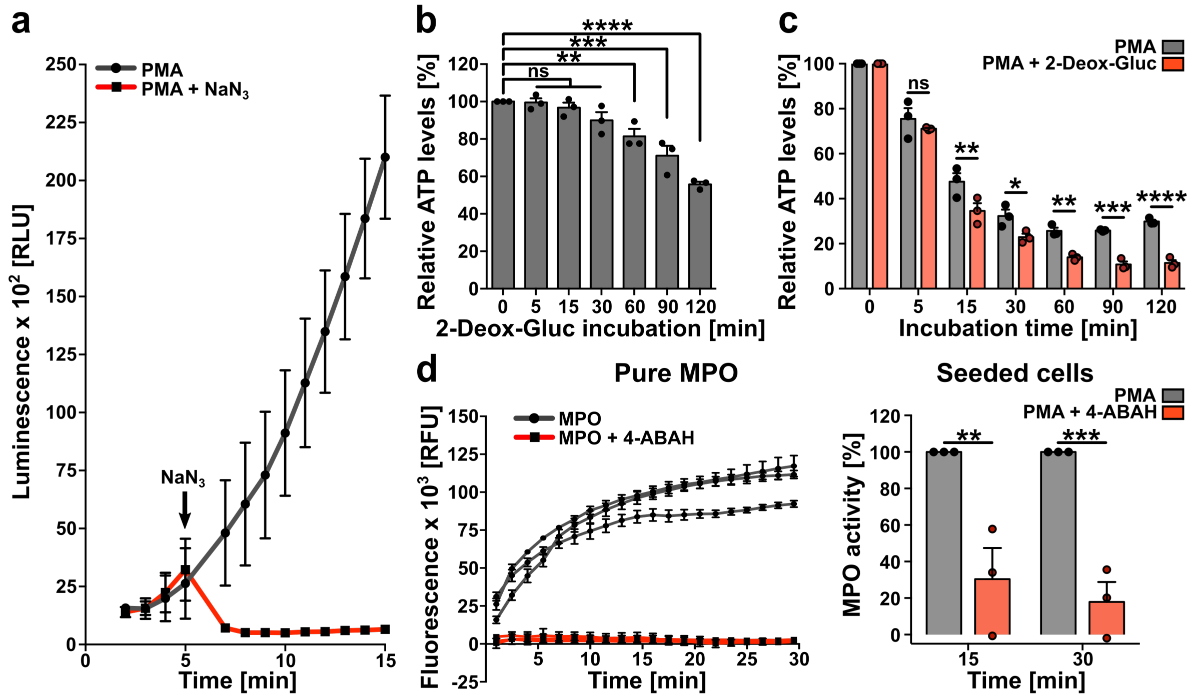

Supplementary Fig. 6: ***Inhibitory effect of NaN_3. 2-Deox-Gluc and 4-ABAH on NETotic cells.*** ***a,*** *Effect of 3 mM sodium azide (NaN_3) on PMA (100 nM)-induced ROS production of human neutrophils determined by chemiluminescence of luminol. NaN_3 inhibits ROS directly after addition and enables a stable inhibition for at least 30 min, as shown in* ***Supplementary Fig. 9c****. Experimental setup comparable with the setup used for the experiments shown in* ***Fig. 3b****. N = 1 (triplicates). Mean ± SD.* ***b****, ATP levels of unstimulated neutrophils after incubation with 5 mM 2-Deoxy-glucose (2-Deox-Gluc) for different time intervals. 2-Deox-Gluc reduces ATP levels already after short incubation and significantly after more than 60 min compared to untreated cells (t = 0 min). Statistics: One-way ANOVA (Bonferroni's multiple comparison test, **p<0.01, ***p < 0.001, ****p < 0.0001). N = 3. Mean ± SEM.* ***c,*** *ATP levels of PMA (100 nM)-stimulated neutrophils with and without incubation with 5 mM 2-Deox-Gluc for different time periods. 2-Doxy-Gluc significantly decreases the ATP levels after more than 15 min PMA stimulation compared to exclusive PMA treatment (t = 0 min). PMA stimulation alone decreases ATP levels by more than 70 %. Experimental setup comparable with the setup used in* ***Fig. 3b****. Statistics: Two-way ANOVA (Bonferroni's multiple comparisons test, *p < 0.05, **p<0.01, ***p < 0.001, ****p < 0.0001). N = 3. Mean ± SEM.* ***d,*** *inhibitory effect of 100 µM 4-aminobenzoic acid hydrazide (4-ABAH) on MPO activity.* ***left****, 4-ABAH inhibits purified MPO significantly and stable after 1 min for at least 30 min. N = 3. Mean ± SD.* ***right,*** *4-ABAH inhibits PMA (100 nM)-induced MPO activity significantly and stable after 15 min and 30 min cell incubation followed by complete cell lysis for MPO activity measurements. Experimental setup comparable with the setup in* ***Fig. 3b****. Statistics: Two-way ANOVA (Bonferroni's multiple comparisons test, **p<0.01, ***p < 0.001). N = 3. Mean ± SEM.*

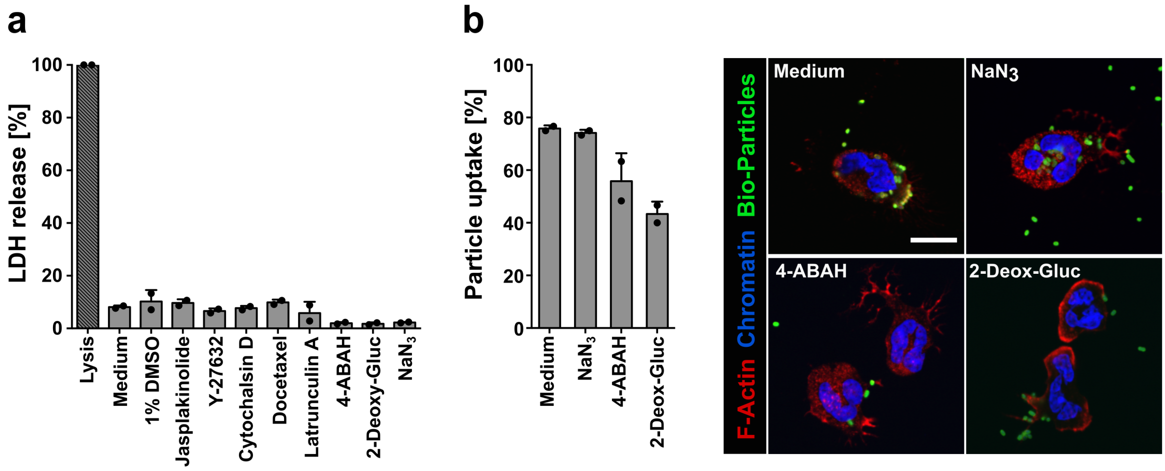

Supplementary Fig. 7: ***Influence of metabolic and cytoskeletal inhibitors on neutrophil function. a****,* *Toxicity of used inhibitors on human neutrophils detected by release of lactatdehydrogenase (LDH) relative to complete cell lysis. All inhibitors were tested for the concentrations used in this study and the maximal incubation time of 3h. All inhibitors show less than 10% cell toxicity, which is below the toxicity of 1% DMSO with 10.2% (maximal solvent concentration in inhibitor studies). N = 2. Mean ± SD.* ***b,*** *Effect of NaN_3 (3 mM), 4-ABAH (100 µM) and 2-Deox-Gluc (5 mM) on uptake of FITC-labeled E. coli BioParticles. The particle uptake is not affected by NaN_3, but clearly decreased in presence of 4-ABAH and 2-Deox-Gluc. Calculation based on confocal imaging of fixed samples after incubation with BioParticles for 30 min. Staining: red = F-actin/Phalloidin555, blue = chromatin/Hoechst, green = FITC-labeled E. coli BioParticles. Scale = 10 µm. N = 2 (n = 60 cells per condition and N). Mean ± SD.*

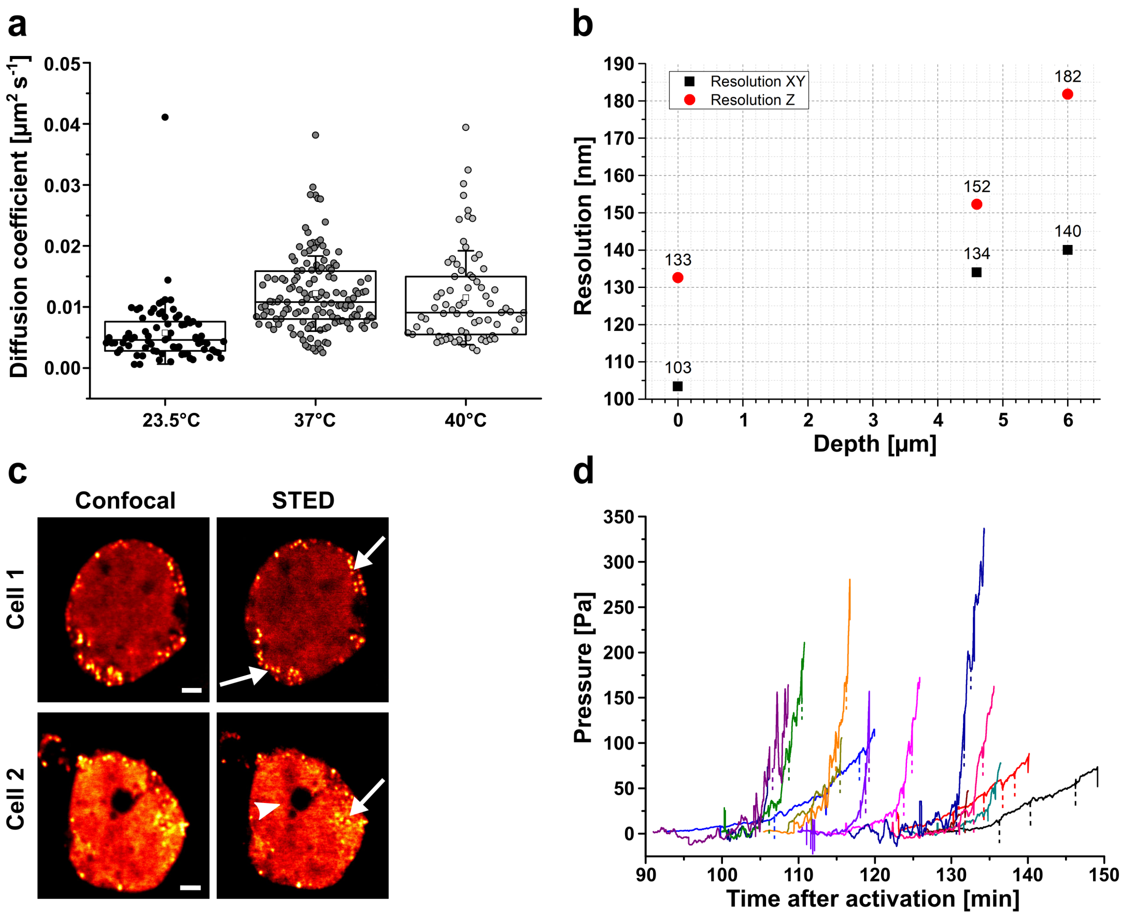

Supplementary Fig. 8*: Properties and consequences of chromatin swelling:* *a,* *Effective 2D diffusion coefficient of chromatin expansion in P2 (t_1 to t_D) at different temperatures (Median: 23.5°C = 0.0047 µm^2 s^{-1}, 37°C = 0.0108 µm^2 s^{-1}, 40°C = 0.0091 µm^2) calculated as described in* ***Supplementary Fig. 3a****. N = 3 (23.5°C, 40°C). N = 5 (37°C). Boxplot displays the 25th and 75th percentile, with the horizontal line at the median, squares represent the mean and whiskers SD.* ***b,*** *Determination of STED-resolution by measuring the size of calibration beads on living neutrophils. The resolution depends clearly on the distance from the coverslip (depth).* ***c,*** *Confocal and STED images of living neutrophils during PMA-induced NET formation (z-direction: around 2-4 µm from the cover slip; cell middle). Chromatin staining: SiR-DNA. Arrow = chromatin dots, mostly appearing close to the surface. Arrowhead = chromatin free areas occurring during the swelling process. Scale = 2 µm. Sometimes in these images small regions of higher intensity (small dots) were observed. It could be chromatin filled vesicles.* ***d****, Pressure curves measured by tipless cantilever AFM experiments. By getting in contact with a PMA activated cell (at least 90 minutes after activation, 100 nM PMA), the cell starts to push on the cantilever resulting in a deflection of the cantilever. Dividing these forces by the cellular contact area, an internal pressure was estimated (see Methods) that likewise increases until the membrane ruptures (end of the traces). Dashed lines are indicating the time points when the deflection values were reset manually to avoid signal loss of the AFM detector. N = 5 donors (n = 14 cells).*

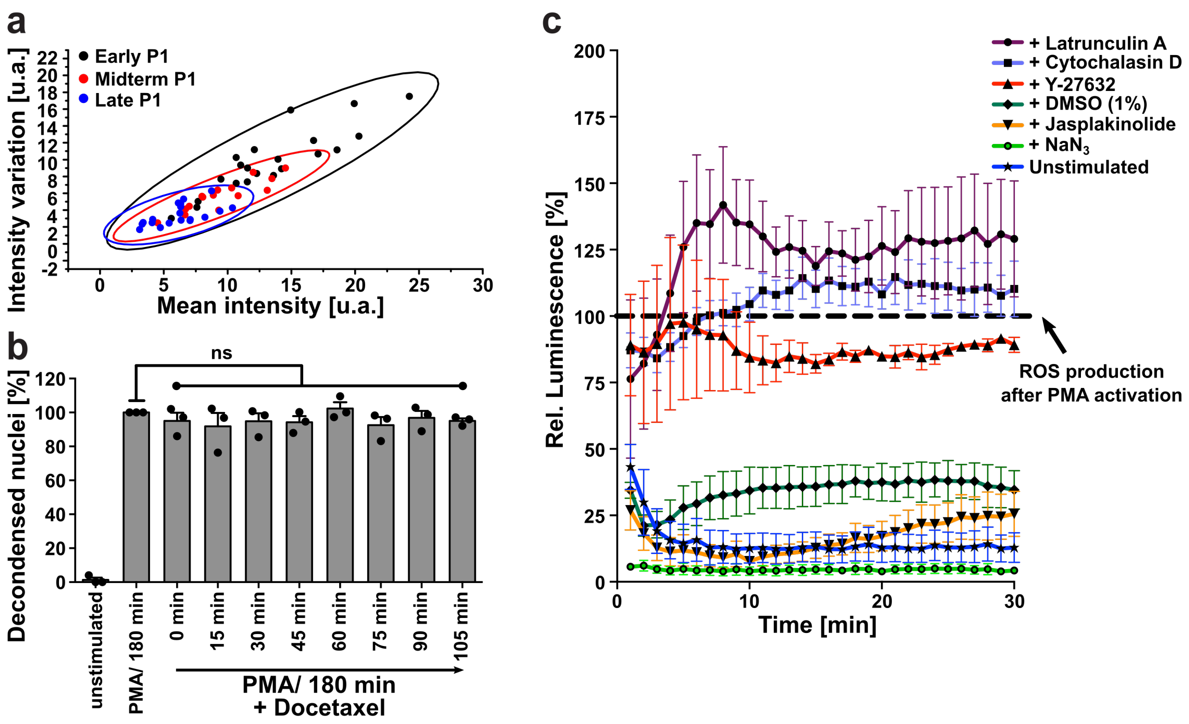

Supplementary Fig. 9: ***Influence of the cytoskeleton:*** ***a,*** *Quantification of F-Actin disassembly. The mean fluorescence intensity of F-actin decreases with time during NETosis. At the same time the heterogeneity decreases, which is a measure for F-actin structure and not biased by bleaching. Thus, F-actin gets disassembled during NETosis. N = 1 donor, data fitted with 95% confidence ellipse.* ***b,*** *NET formation after treatment with Docetaxel (100 nM, inhibition of tubulin depolymerization) at different time points after induction of NETosis with PMA. Docetaxel shows no influence on NET formation (measured as %-relative number decondensed nuclei after 180 min compared to exclusive activation with PMA). n = 3. Mean ± SEM. ns = not significant. Statistics: One-way ANOVA (Bonferroni's multiple comparison test)* ***c,*** *Influence of actin cytoskeletal inhibition on PMA (100 nM)-induced ROS production of human neutrophils determined by the chemiluminescence of luminol. Latrunculin A (violet) increases ROS, while Cytochalasin D (violet) and Y-27632 (red) have no or only slight effects on ROS production in the concentrations used for the experiments shown in* ***Fig. 5b, c****. In contrast, Jasplakinolde (yellow) shows a strong inhibitory effect. As controls, the ROS levels of unstimulated cells (blue), cells after addition of 1% DMSO (dark green, used for Jasplakinolide experiments) and NaN_3 (green) are shown. N = 3. Mean ± SEM.*

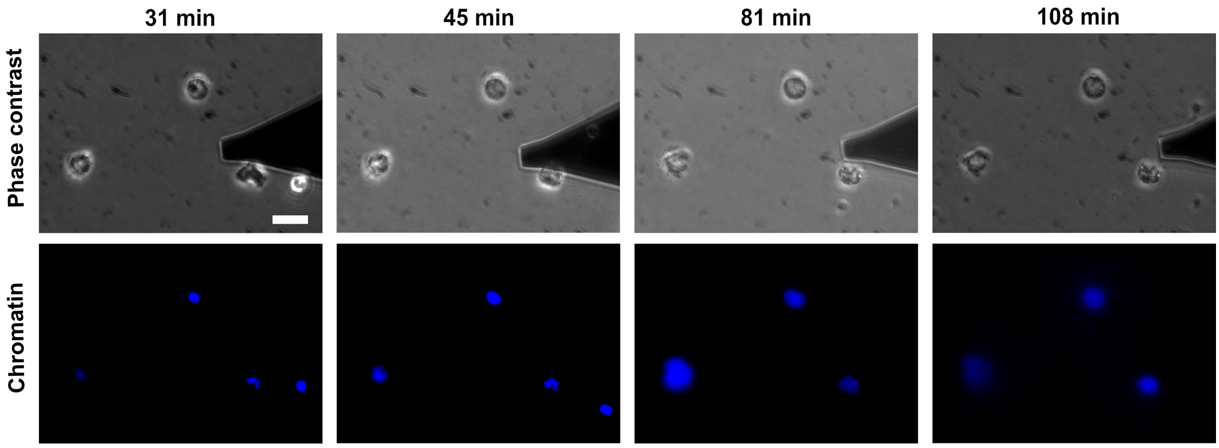

Supplementary Fig. 10: ***Morphology of neutrophils during AFM.*** *Phase contrast and chromatin (blue) images of activated neutrophils during life cell AFM experiments. Within the early stage of NETosis, lobular shape of the nucleus and cell rounding can be observed. After around 40 minutes, DNA decondenses und the cells rupture around t = 80-110 min. No morphological difference between cells probed by AFM and control cells can be observed. Scale = 20 µm.*

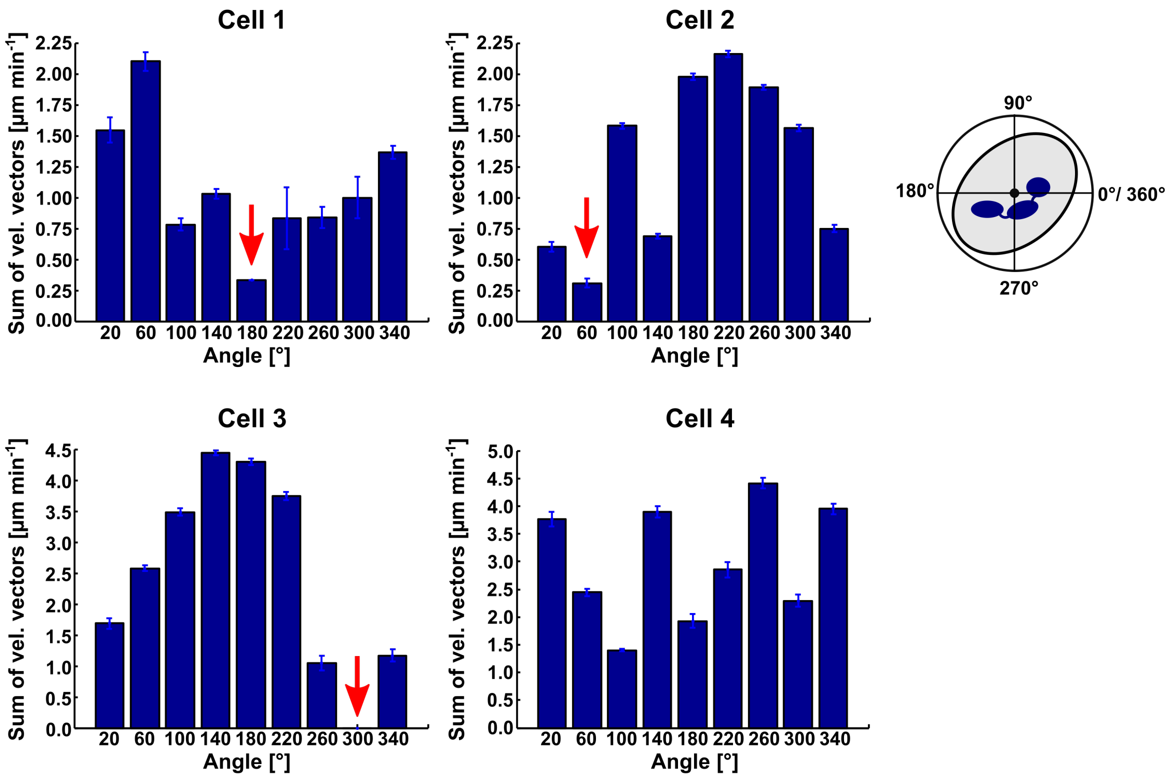

Supplementary Fig. 11: ***Chromatin swelling directs location of membrane rupture.*** *Chromatin swelling analysis (data of the cells shown in **Fig. 6a)**. To analyze whether the position of the rupture point correlates with the chromatin swelling behavior, the average swelling velocity and direction was calculated and visualized in a velocity plot (see also Methods). These velocity vectors were sorted (binning angle = 40°) and added up to find regions of different swelling behaviors. Regions of low velocities (red arrow, green circle in **Fig. 6a**) are often in close proximity to the observed rupture point position (red circle in **Fig. 6a**, cell 1-3), whereas no correlation is detectable for uniform swelling processes (cell 4). In this case, the nucleus is centered in the cell. Bar plot show the sum of all velocity vectors within one bin (vector length of all summed up velocity vectors). Error bars display SEM of the aforementioned vectors.*

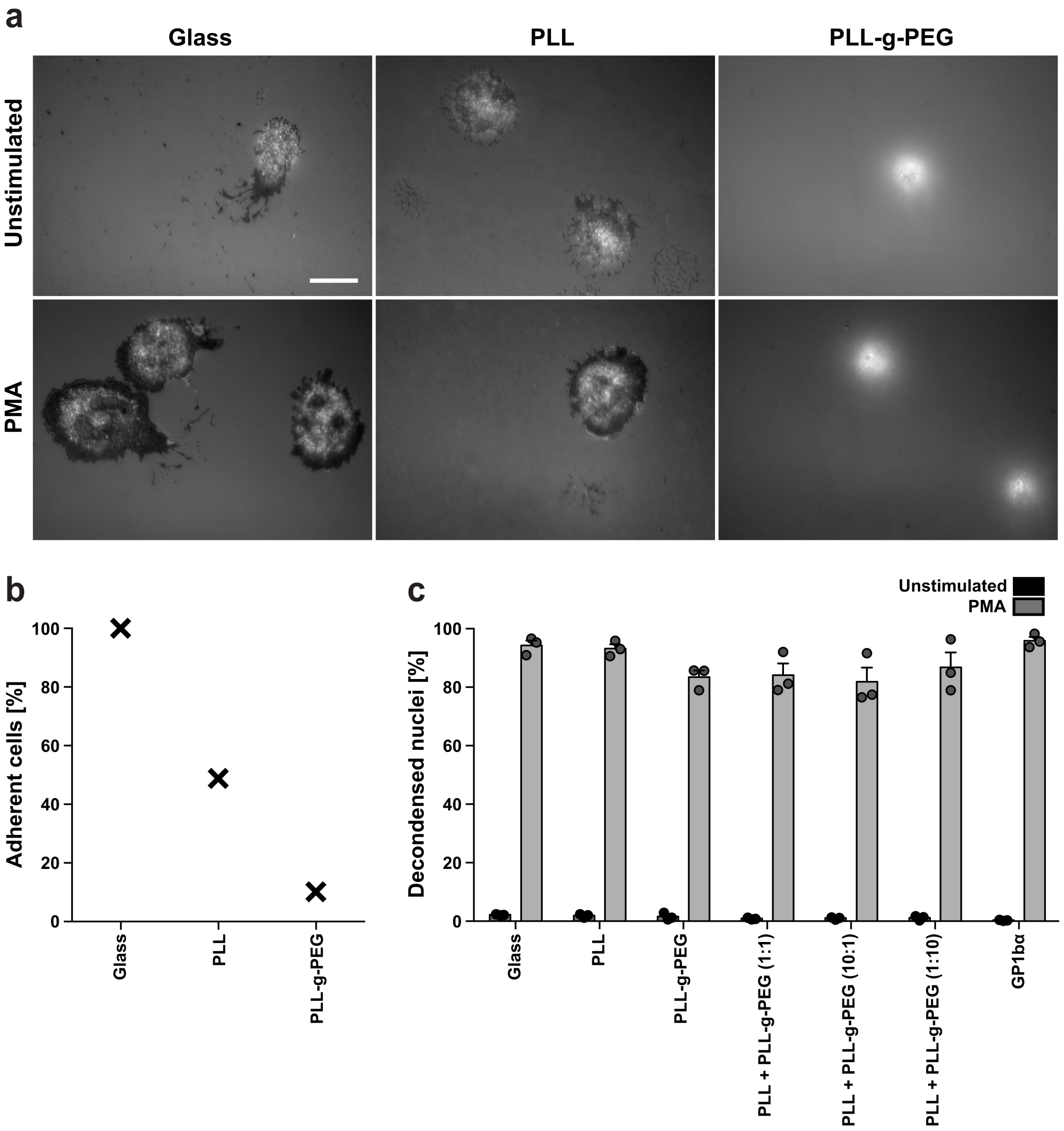

Supplementary Fig. 12: ***PMA-induced NETosis is independent of adhesion. a,*** *RICM images of fixed neutrophils on glass after one-time washing. Images show cells incubated with PMA for 30 min or cells left unstimulated. Cells on PLL-g-PEG (Poly-L-Lysine-grafted-PolyEthylenGlycole) coated surfaces are barely adherent compared to neutrophils on glass or PLL coating. Scale = 10 µm.* ***b,*** *Cell numbers after one-time washing of cells seeded for 30 min on different surfaces. In PLL-g-PEG coated wells only a few cells remain, compared to glass and PLL coating. These cells are still barely adherent as shown in* ***a****. N = 1.* ***c,*** *PMA-induced NETosis (100 nM) performed on different surfaces. The amount of decondensed nuclei is independent of the surface/adhesive properties (surfaces of different passivation level, GPIbα is the ligand of Mac-1 integrin). No washing step was included in this procedure. N = 3. Mean ± SEM.*

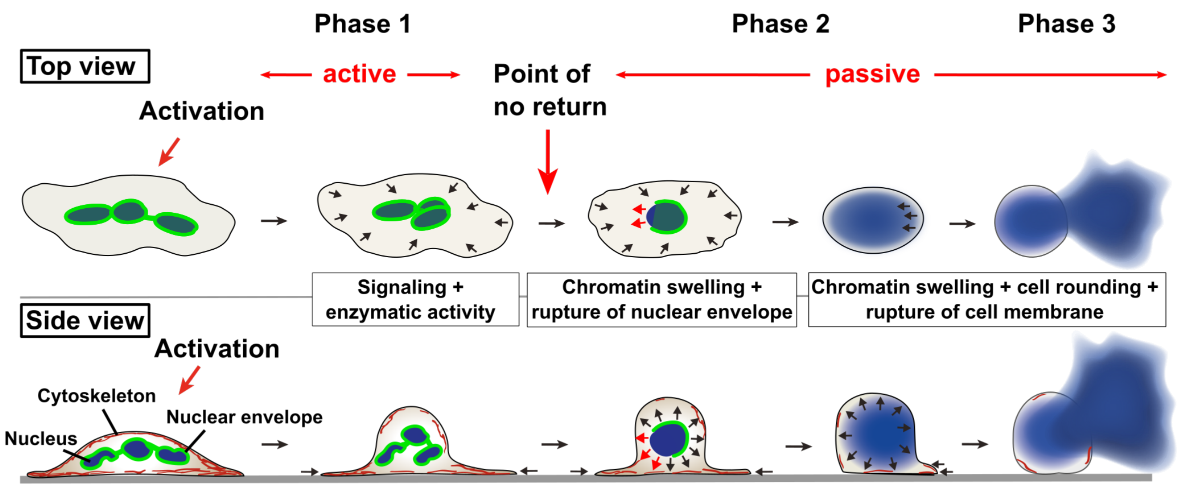

Supplementary Fig. 13: ***Detailed biophysical model of NET formation.***

Supplementary Methods

Surface coating

For platelet glycoprotein 1b alpha chain (GP1bα) coating, 96-glassbottom-well-plates were incubated over night with 5 μg ml^{-1} GP1bα (R&G Systems) at 4°C. Then, the wells were washed with 1x PBS followed by blocking with 3% BSA (Roth) for 2 h at 37°C and an additionally washing step.

For Poly-L-lysine hydrobromide (PLL, Sigma-Aldrich) and poly(L-lysine)-graft-poly(ethylene glycol) (PLL-g-PEG, SuSoS AG) coating, 96-glassbottom-well-plates were incubated either with 0.5 mg mL^{-1} PLL in 10 nM HEPES or 0.5 mg mL^{-1} PLL-g-PEG in 10 nM HEPES at room temperature for 45 min. Then solutions were removed and washed one time with culture medium. Further cell experiments were carried out as described below.

Adhesion experiments

Fresh isolated human neutrophils (10 000 per well) were seeded in 96-glassbottom-well-plates directly or wells coated with GP1bα, PLL and/or PLL-g-PEG (0:1, 1:10, 1:1, 10:1, 1:0) as described above. Then, cells were activated for NETosis with PMA in a final concentration of 100 nM for 3h (37 °C, 5% CO_2). All experiments were performed in triplicates. To end NET formation, cells were fixed with 2% PFA final concentration and stored over night at 4°C. The fixed probes were stained with 1.62 μM Hoechst 33342 and the percentage of decondensed nuclei/NETs was analyzed as described in the section Inhibitor Experiments in Methods.

To analyze the adhesion on the different surfaces, 10 000 neutrophils per well were seeded in 96-glassbottom well-plates coated with PLL or PLL-g-PEG or left untreated. The cells were washed one time with 1xPBS after 30 min incubation (37 °C, 5% CO_2) with or without PMA (100 nM). Then, cells were fixed with 2% PFA final concentration. Subsequently, the cells were stained with Hoechst and the amount of remaining cells calculated relatively to the glass sample using ImageJ (six images/well counted in defined regions, counting blinded). Representative RICM images of the fixed samples were recorded in the setup described in the section Reflection Interference Contrast Microscopy (RICM) in Methods.

For time-lapse observations, 4 x 10^5 cell per ml were seeded in an ibidi treat flow chamber coated with PLL-g-PEG and the movie recorded as described in the section Live Cell Imaging" in Methods.

Reflection Interference Contrast Microscopy (RICM)

For RICM, ibidi treated petri dishes (Ibidi GmbH) with a 35 mm radius glass bottom (81158, ibidi) or ibidi treat flow chambers uncoated or coated with PLL were used. 100 000 neutrophils per petri dish or 4 x 10^5 cells per ml per flow chamber were seeded and stained with Hoechst. The microscope setup contained a heating chamber (ibidi heating system, ibidi GmbH) on top of the microscope stage (Axiovert 200, Zeiss) and the temperature was adjusted to 37 C° for all experiments. NETosis was induced by 100 nM PMA. Time-lapse movies were recorded (1 frame per min) 63x magnified (EC Plan-Neofluar Ph3 objective/420481-9911-000, 1.6x Optovar, Zeiss) for both DAPI as well as RICM

images. Samples were illuminated by a XCite Series 120Q or HBO 100 (1007-980, Zeiss) lamp together with a respective DAPI (Filter set 02 shift free/488002-9901-000, Zeiss) or RICM filter set (reflector module Pol ACR P&Cfor HBO 100/424924-9901-000, emission filter 416 LP, AHF-Nr.: F76-416/000000-1370-927, Zeiss) and observed with a Zyla SCMOS camera (AndorZyla 5.5) using Micro Manager software (v.1.4) or a CoolSNAP ES camera (Photometrics) using the software Metamorph 6.3r2. (Molecular Devices Inc.). Subsequently, the image contrast was adjusted with ImageJ.

ATP measurements/ 2-Deox-Gluc

Fresh isolated human neutrophils (10 000 per well in RPMI (10 mM HEPES, 0.5% HSA, without phenolred) were seeded in white 96-well-plates and metabolic activity inhibited by adding 5 mM 2-Deox-Gluc for defined time periods at 37°C (5 min, 15 min, 30 min, 60 min, 90 min and 120 min). Simultaneously, cells were activated with PMA in a final concentration of 100 nM or left untreated. After incubation the ATP amount was measured as described in the section ATP measurements in Methods. All experiments were carried out in triplicates and ATP levels were calculated relatively to the ATP amount of unstimulated cells incubated for 120 min without addition of 2-Deox-Gluc.

MPO activity

The activity of MPO was measured with the myeloperoxidase chlorination fluorometric assay kit from Cayman chemical following the company's instructions. To prove the inhibitory effect of 100 μM 4-ABAH on MPO, two different setups were used. First, the effect on purified MPO supplied by the company was studied in presents or absents of 4-ABAH. The activity of MPO was determined by the formation of fluorescein (Thermo Scientific APPLISKAN®, Software: Skanlt RE for Appliskan 2.3, Thermo Fisher Scientific) at 485 nm/535 nm over 29.5 min (frame rate: 1.5 min) at room temperature. All experiments were performed in triplicates. Second, human neutrophils (1 000 000 per well in RPMI (10 mM HEPES, 0.5% HSA)) were seeded in 24-well plates and activated with 100 nM PMA in the presents or absents of 4-ABAH for 15 min or 30 min at 37°C, respectively. After incubation, the culture medium was removed and cells were washed with 1x PBS. Then, the cells were scraped from the plate and pelleted by 1000 g for 10 min at 4°C. Cell lysis was induced by ultra shall sonication in combination with freeze-thraw cycles. After lysis, cell remnants were removed by centrifugation at 10 000 g for 10 min at 4°C and MPO activity in the supernatant was determined as described above. For all conditions the change in fluorescence signal between 1 min and 29.5 min was determined and the relative MPO activity was calculated between 4-ABAH treated and untreated cells.

Reactive oxygen species (ROS) measurement

Fresh isolated human neutrophils (10 000 per well in HBSS (10 mM HEPES, without phenolred) were seeded in white 96-well-plates (Greiner bio-one) at 37°C. After seeding, luminol (Sigma-Aldrich) was added at a final concentration of 60 μM and actin cytoskeletal components were inhibited by Cytochalasin D (100 nM), Latrunculin A (1 μM), Jasplakinolide (10 μM) or Y-27632 (19.2 μM)

respectively. Directly after addition of inhibitors, NETosis was activated with 100 nM PMA and the luminescence was measured (GLOMAX® 96 Microplate Luminometer, Software: GLOMAX 1.9.3, Turner BioSystems) at room temperature (frame rate: 1 min). As controls the change in luminescence signal was recorded for unstimulated cells, cells treated with the maximal solvent concentration of 1% DMSO (used in Japlakinolide studies) and cells which were inhibited with 3 mM NaN_3. All experiments were carried out in triplicates and the luminescence signal was determined relatively to ROS levels after exclusive PMA activation.
For evaluation of NaN_3 activity, cells were seeded (90 000 per well) and activated as described above. NaN_3 (3 mM) was added 5 min after activation with PMA and the change in the luminescence signal was continuously recorded.

Cell toxicity

Cell toxicity of cytoskeletal and metabolic inhibitors was measured by the release of lactatdehydrogenase (LDH) with the CytoTox 96® Non Radioactive Cytotoxicity Assay (Promega) as instructed by the company. By analogy with the assays described in the section Inhibitor Experiments in Methods, 10 000 cells were seeded in plastic 96-well plates (Greiner bio-one) and incubated in RPMI (10 mM HEPES, 0.5% HSA, without phenolred) with Cytochalasin D (100 nM), Latrunculin A (1 μM), Docetaxel (100 nM), Jasplakinolide (10 μM), Y-27632 (19.2 μM), 2- Deox-Gluc (5 mM), NaN_3 (3 mM), 4-ABAH (100 μM), DMSO (1%) or only culture medium for 3 hours at 37°C. The released LDH was measured in the supernatant as described in the assay protocol and cell toxicity was calculated relatively to maximal cell lysis.

Uptake of particles

Fresh isolated human neutrophils (100 000 per well in RPMI (10 mM HEPES, 0.5% HSA) were seeded on pretreated (99% alcohol) glass cover slips (#1.5) in 24-well plates followed by pre-incubation for 30 min with or without 2- Deox-Gluc (5 mM), NaN_3 (3 mM) or 4-ABAH (100 μM), respectively. Then, cells were further incubated with fluorescein-labeled Escherichia coli (K-12 strain) BioParticles® (0.1 mg ml^{-1}, Vybrant™Phagocytosis Assay Kit (V-6694), Thermo Fisher Scientific) in presents of 2- Deox-Gluc (5 mM), NaN_3 (3 mM) or 4-ABAH (100 μM) and incubated for additionally 30 min. For fixation, 2% PFA final concentration was added and samples then stored after washing with 1x PBS. Cells were stained as described in the section Staining Procedure in Methods for F-actin and chromatin with Phalloidin (PromoFlpur-555P, PromoKine) and Hoechst directly after blocking. For each condition the particle uptake was analyzed manually by 3D confocal laser scanning microscopy (60x magnified, Olympus IX83 inverted microscope, software: Olympus Fluoview v.4.2) and the percentage of particle uptake calculated relatively to the total cell count of 60 cells per condition.

Quantification of fluorescence imaging

For the quantification of Lamin B1 rupture, the rupture of the nuclear envelope was determined for different time points after induction of NETosis with PMA. Therefore, cells were stained for Lamin B1 and Chromatin as described in the section Staining Procedure in Methods and the nuclear envelope rupture was analyzed manually by 3D confocal laser scanning microscopy (60x magnified, Olympus IX83 inverted microscope, software: Olympus Fluoview v.4.2). For each condition the loss of the nuclear envelope (small rupture up to complete loss) was determined for 100 cells and calculated relatively to the total cell count.

Similarly, to quantify the loss of actin within phase 1, CLSM images of stained F-actin areas of fixed cells (exemplarily shown in **Fig. 5a**) where analyzed using the ImageJ thresholding plugin. More precisely, within all pictures, the F-actin area of individual cells were segmented first (Yen thresholding method) and the intensity values of the enclosed actin area was averaged to obtain both, a mean intensity value as well as a number for the intensity variation within the respective area (standard deviation). Both values were plotted to visualize the correlation between the amount and the homogeneity of actin within P1 and a 95% confidence ellipse was drawn.
