# Supplementary material for: Chromatin swelling drives neutrophil extracellular trap release
Source: Nat Commun. 2018 Sep 14;9:3767. doi: 10.1038/s41467-018-06263-5 (PMC6138659; doi:10.1038/s41467-018-06263-5)
Supplement: Supplementary file 3 — Description of Additional Supplementary Files [file 41467_2018_6263_MOESM3_ESM.pdf]

Description of Additional Supplementary Files

File Name: Supplementary Movie 1
Description: Time-lapse confocal laser scanning microscopy (CLSM) movie of human neutrophils undergoing NETosis stimulated with 100 nM PMA. Cells are stained for chromatin (blue, Hoechst 33342) and membrane (red, PKH26).

File Name: Supplementary Movie 2
Description: Neutrophil during PMA-induced NET formation (example cell in Fig. 1a and 1b).

File Name: Supplementary Movie 3
Description: Time-lapse movie of chromatin decondensation of human neutrophils observed with conventional fluorescence microscopy during PMA-induced NET formation. Chromatin stained by Hoechst 33342.

File Name: Supplementary Movie 4
Description: Example cell 1 of Supplementary Movie 3.

File Name: Supplementary Movie 5
Description: Example cell 2 of Supplementary Movie 3.

File Name: Supplementary Movie 6
Description: Example cell 3 of Supplementary Movie 3.

File Name: Supplementary Movie 7
Description: Time-lapse movie of chromatin decondensation of neutrophils activated to undergo NET formation by PMA (100 nM) (left), LPS (25 µg ml^{-1}, middle) or CaI (4 µM, right), respectively. Chromatin stained by Hoechst 33342.

File Name: Supplementary Movie 8
Description: Time-lapse 3D-CLSM movie of human neutrophils undergoing NETosis stimulated by 100 nM PMA. Cells are stained for chromatin (blue, Hoechst 33342) and membrane (red, PKH26). z-Stack-depth = 1 µm, 10 minutes/frame.

File Name: Supplementary Movie 9
Description: Reflection interference contrast microscopy (RICM) time-lapse movie on a glass substrate (Supplementary Fig. 4c). Neutrophils are activated with 100 nM PMA.

File Name: Supplementary Movie 10
Description: RICM-time-lapse movie on an ibidi treat substrate (surface equal to CLSM time-lapse movies). Neutrophils are activated with 100 nM PMA.

File Name: Supplementary Movie 11
Description: RICM-time-lapse movie on an ibidi treat substrate coated with Poly–L-lysine (PLL). Neutrophils are activated with 100 nM PMA.

File Name: Supplementary Movie 12
Description: Time-lapse movie of chromatin decondensation of neutrophils activated to undergo NET formation by PMA (100 nM) at 23.5°C (left), 37°C (middle) or 40°C (right), respectively. Chromatin stained by Hoechst 33342.

File Name: Supplementary Movie 13
Description: Example cell 1 in Fig. 2d (cells from Supplementary Movie 1).

File Name: Supplementary Movie 14
Description: Example cell 2 in Fig. 2d (cells from Supplementary Movie 1).

File Name: Supplementary Movie 15
Description: Example cell 3 in Fig. 2d (cells from Supplementary Movie 1).

File Name: Supplementary Movie 16
Description: Time-lapse movie of a typical height (left) and stiffness map (right) during life cell AFM measurement. The raw data of the marked pixel (force curve) is shown below for each frame.

File Name: Supplementary Movie 17
Description: Time-lapse movie of chromatin decondensation during PMA-induced NET formation on a poly(L-lysine)-graft-poly(ethylene glycol) (PLL-g-PEG) coated ibidi treat substrate (right) in comparison to only ibidi treat (left, conventional fluorescence microscopy). Chromatin stained by Hoechst 33342.
